# Ulcer of the Rectum Operated On

**Published:** 1831

**Authors:** 


### XXXV.

### Ulcer of the Rectum operated on.

In the Med. and Physical Journal, for April, there is a case of the above kind reported from the Middlesex Hospital, which is worthy of notice. The patient was a female, aged 38, who, for two years previously had experienced pain in the rectum, when the bowels were evacuated. This increased in severity, and was attended by unpleasant sensations about the loins, &c. and some sanguineous discharge. In December, 1830, she entered the Middlesex, labouring under severe pains in the rectum, with the occasional issue of pus and blood.

On examination of the rectum, it was found to be indurated and ulcerated to the extent of two inches ; but the finger could be passed beyond the diseased part into a healthy gut. Various remedies were tried, but in vain. Mr. Mayo then determined on an operation.

"The operation was performed on the 25th of February, in the following manner:—the patient was laid upon her side, with the hips and knees bent. The fingers being then introduced into the rectum, the knife was plunged into the perineum, on one side of the bowel, and, an incision of some depth being thus made laterally, the dissection was continued forwards from thence, so as to separate the vagina from the rectum. The dissection was then continued entirely round the rectum, including half an inch of integument, with the sphincter muscle. By this means, a length of two inches and a half of the extremity of the rectum was separated from the adjacent parts : it was then cut off with scissors from the sound rectum above. The operation was performed slowly, and the vessels, about nine in number, were tied as they were divided. The patient lost about twelve or fourteen ounces of blood."

In about two hours after the operation, and when the smarting of the wound had subsided, she observed that she found herself entirely relieved from the pain and distress to which she had been subject for so many months. The appearance of the wound is singular. The extremity of the bowel is not more than half an inch from the cut edge of the skin, and the intervening granulations are healthy and rapidly cicatrizing. The bowels act regularly once a day ; and the patient is aware of the presence of the fæces in the rectum. In about five minutes after this sensation is perceived, the bowels act much in the usual manner, though it is evident that there can be nothing at present equivalent to a sphincter muscle. A hope is entertained that when the wound is cicatrized and contracted, the patient will have some power of retaining solid fæces.

It is probable that the above is the first operation of the kind, or at least to the same extent, which has been performed in this country. We know of one case where a medical gentleman had a chronic and painful ulcer, surrounded with raised and indurated edges, at about an inch from the extremity of the rectum. Mr. Guthrie dissected it out. Several abscesses formed, and two sinuses were afterwards obliged to be laid open in the same manner as for fistula. The sphincter was, of course, divided, and part of it removed in this operation ; but the patient has entirely recovered, and the function of the sphincter is as perfect as ever.

---

